# Construction of Cu_2_Y_2_O_5_/g-C_3_N_4_ Novel Composite for the Sensitive and Selective Trace-Level Electrochemical Detection of Sulfamethazine in Food and Water Samples

**DOI:** 10.3390/s24175844

**Published:** 2024-09-09

**Authors:** Rajendran Surya, Subramanian Sakthinathan, Ganesh Abinaya Meenakshi, Chung-Lun Yu, Te-Wei Chiu

**Affiliations:** 1 Department of Materials and Mineral Resources Engineering, National Taipei University of Technology, No. 1, Section 3, Chung-Hsiao East Road, Taipei 106, Taiwan; suryarajendran844@gmail.com (R.S.); abimeenakshi1998@gmail.com (G.A.M.); samweo909li@gmail.com (C.-L.Y.); 2 Institute of Materials Science and Engineering, National Taipei University of Technology, No. 1, Section 3, Chung-Hsiao East Road, Taipei 106, Taiwan

**Keywords:** sulfamethazine, transition metal oxide, electrochemical sensor, limit of detection, cow milk samples

## Abstract

The most frequently used sulfonamide is sulfamethazine (SMZ) because it is often found in foods made from livestock, which is hazardous for individuals. Here, we have developed an easy, quick, selective, and sensitive analytical technique to efficiently detect SMZ. Recently, transition metal oxides have attracted many researchers for their excellent performance as a promising sensor for SMZ analysis because of their superior redox activity, electrocatalytic activity, electroactive sites, and electron transfer properties. Further, Cu-based oxides have a resilient electrical conductivity; however, to boost it to an extreme extent, a composite including two-dimensional (2D) graphitic carbon nitride (g-C_3_N_4_) nanosheets needs to be constructed and ready as a composite (denoted as g-C_3_N_4_/Cu_2_Y_2_O_5_). Moreover, several techniques, including X-ray diffraction analysis, scanning electron microscopy analysis, energy-dispersive X-ray spectroscopy, Fourier transform infrared spectroscopy, and Raman spectroscopy were employed to analyze the composites. The electrochemical measurements have revealed that the constructed g-C_3_N_4_/Cu_2_Y_2_O_5_ composites exhibit great electrochemical activity. Nevertheless, the sensor achieved outstanding repeatability and reproducibility alongside a low limit of detection (LOD) of 0.23 µM, a long linear range of 2 to 276 µM, and an electrode sensitivity of 8.86 µA µM^−1^ cm^−2^. Finally, the proposed GCE/g-C_3_N_4_/Cu_2_Y_2_O_5_ electrode proved highly effective for detection of SMZ in food samples, with acceptable recoveries. The GCE/g-C_3_N_4_/Cu_2_Y_2_O_5_ electrode has been successfully applied to SMZ detection in food and water samples.

## 1. Introduction

Sulfamethazine (SMZ), frequently referred to as 4-amino-n-(4, 6-dimethylpyrimidin-2-yl) benzene sulfonamide, pertains to the sulfonamide family, a widely used class of veterinary medicine, antimicrobial agents, and a preventative measure. In recent times sulfamethazine has gained quite an impressive amount of interest because of being one of the essential antibacterial medications for treating several illnesses, including urinary tract infections, chancroid, *Toxoplasma gondii* encephalitis, etc. [1]. However, SMZ overdose or prolonged exposure in the human body can lead to severe chronic toxicity, inflammation of the liver, and genetic disruption, which could boost death and disability rates [2]. Moreover, excess consumption of SMZ tends to stimulate carcinogenesis, tetra genesis, and mutagenesis. For this reason, it is crucial to find SMZ in consumables at trace levels [3]. For this reason, we have developed a precise method for detecting SMZ. There are enormous methods to detect SMZ compared to other analytic methods like capillary electrophoresis [4], UV spectrophotometry, and biosensors [5]. These aforementioned techniques are used to analyze SMZ on food samples. Moreover, the detection of SMZ in animal fluids and tissue detection methods include gas chromatography, chemical ionization, electron-capture or flame ionization detection, immunoassay, calorimetric, enzyme-linked immunosorbent analysis, and screening for microbial diffusion. Most of these approaches involve a time-consuming, expensive, and labor-intensive sample preparation process; nevertheless, their sensitivity and specificity are low [6,7,8]. In this regard, electrochemical approaches provide advantages such as enhanced sensitivity, user-friendliness, and fully portable instrumentation [9]. In recent times, transition metal oxides have attracted many researchers because of their excellent ability in energy conversion, energy storage, and sensing applications. In addition, copper yttrium oxide (Cu_2_Y_2_O_5_) components [10] are used for solid-state galvanic cells, superconducting materials, p-type semiconductor material, and photocatalytic, and antibacterial properties against *E. coli* in a dark environment. Moreover, the high photocatalytic activity of the Cu_2_Y_2_O_5_ component applied to hydrogen evolution because of good electron transfer, high surface area, thermal stability, catalytic activity, electromagnetic properties, and layered structure of the Cu_2_Y_2_O_5_ component. In addition, the as-prepared Cu_2_Y_2_O_5_ has unique size-dependent properties. Therefore, based on the aforementioned properties, we have used the Cu_2_Y_2_O_5_ component for the electrochemical sensor application [11,12,13,14]. However, the material shows good sensing performance but it does not exhibit prolonged ability to enhance its stability; therefore, we have to make a composite consisting of graphitic carbon nanosheets (g-C_3_N_4_). However, compared to graphene, g-C_3_N_4_ has a large active area and unique porous networks [15,16,17]. For instance, g-C_3_N_4_ actively modifies the electrical characteristics, making it a suitable material for anchoring dynamic mixed-metal oxide species that enhance electrochemical stability and selectivity [18,19,20,21,22]. Consequently, the construction of Cu_2_Y_2_O_5_ anchored g-C_3_N_4_, which has many electroactive sites and remarkable endurance towards the determination of SMZ, making it ideal for use in sensing applications [23].

Enhanced electron-transfer rates, higher current, and reduced overpotential are accomplished through the utilization of nanomaterials with greater surface areas. This leads to enhanced electrodes that are more resilient, selective, sensitive and have higher electrocatalytic activities [24]. Herein, we have established a new strategy for the unique design of Cu_2_Y_2_O_5_ particles anchored with g-C_3_N_4_ nanosheets for non-enzymatic SMZ sensors. To obtain better sensing capabilities, the highly conductive g-C_3_N_4_ nanosheets will quicken the movement of electrons to the Cu_2_Y_2_O_5_. In particular, the g-C_3_N_4_/Cu_2_Y_2_O_5_ composite displays good interaction between Cu_2_Y_2_O_5_ and g-C_3_N_4_, improving the kinetics of electron transport and the active sites. The elevated electron movement occurs due to a low resistivity and the proposed electrocatalysts’ wide surface area. The aromatic amine group (-NH_2_) electro-oxidation occurs irreversibly in SMZ, reducing the overpotential +0.85 V in comparison with the bare electrode and forming a hydroxylamine group (-NHOH) via the movement of 2H^+^ and 2e^−^ ions in the g-C_3_N_4_/Cu_2_Y_2_O_5_ modified electrode area. The ideal g-C_3_N_4_/Cu_2_Y_2_O_5_ composite has more incredible sensing towards SMZ, featuring outstanding reproducibility, ultralong stability, limit of detection (LOD) of nanomolar (nM) levels, linear detection range, great selectivity, and sensitivity. Compared to the most efficient nonenzymatic sulfamethazine sensors mentioned in the literature, our recently created sensors perform better in terms of sensing. Moreover, effectiveness has been demonstrated by an adequate recovery rate for SMZ detection in real samples with impressive electrochemical sensing characteristics.

## 2. Materials and Methods

### 2.1. Chemical and Reagents

Materials and methods should be described with sufficient details to allow others to replicate, and build on the results. The required chemicals copper nitrate [Cu(NO_3_)_2_⋅3H_2_O], yttrium nitrate [Y(NO_3_)_3_⋅6H_2_O], sodium hydroxide (NaOH), sulfamethazine (C_12_H_14_N_4_O_2_S or SMZ), potassium chloride (KCl), monosodium hydrogen phosphate (NaH_2_PO_4_), disodium hydrogen phosphate (Na_2_HPO_4_), and dimethylformamide (DMF) were purchased from Sigma-Aldrich, Fisher chemical company, Taiwan. Ultrapure deionized water (DI) was used for making the stock solution. Furthermore, to detect sulfamethazine, 0.1 M phosphate buffer solution was employed. Each investigation utilized this solution for a real assessment of the sample. Hence, in the real sample analysis, we collected Tamsui River water from the area around Taipei, Taiwan (25°04′04.4″ N 121°32′15.8″ E) and purchased the milk and honey from the Binjiang local market (25°04′04.4″ N 121°32′15.8″ E) in Taipei, Taiwan.

### 2.2. Characterization Techniques

The configuration of the samples was examined by X-ray diffraction analysis (XRD) (Bruker XRD, D2 Phaser, Billerica, MA, USA, λ = 1.540 Å). After the XRD studies, the data were analyzed using MDI JADE5.0 software. The prepared materials’ surface properties were analyzed by Fourier transform infrared spectrometry (Model: Jasco FT-IR-4600). Raman spectroscopy (DXR Raman spectrometer, Thermo Scientific, Waltham, MA, USA) was used to analyze the Raman shift and symmetry variations. Field emission scanning electron microscopy (FE–SEM, FEI Quanta FEG 200, H-7600, Hitachi, Tokyo, Japan) operating at 200 kHz was used to analyze the morphological information. The electrochemical analysis and electrochemical impedance (EIS) were evaluated by the CHI 1211B workstation, these CHI 1211B workstations (CH Instruments, Inc., Austin, TX, USA) were linked to a three-electrode electrochemical cell system, which included an Ag/AgCl electrode as a reference electrode, a GCE as a working electrode, and a Pt needle as a counter electrode (BAS Inc., Tokyo, Japan). All the electrochemical experiments were conducted at room temperature in a N_2_-purged electrolyte atmosphere.

### 2.3. Synthesis of Cu_2_Y_2_O_5_ Powder

The Cu_2_Y_2_O_5_ powder was synthesized by following the previous methods [25,26]. In this process, 0.01 M copper nitrate [Cu(NO_3_)_2_⋅3H_2_O] and 0.01 M yttrium nitrate [Y(NO_3_)_3_⋅6H_2_O] were dispersed in purified DI water to obtain a homogenous mixture. The mixture was stirred vigorously to obtain a precursor solution without any precipitates. Then glycine (C_2_H_2_NO_2_) was added to the resulting metal nitrate solution and the mixture was stirred at 80 °C for several hours. The glycine-to-metal ratio was estimated at 1.7. The final mixture was kept in a hot air oven to evaporate water and then spontaneously combusted at 350 °C to yield a Cu_2_Y_2_O_5_ powder. This Cu_2_Y_2_O_5_ powder was annealed at 700 °C in the N_2_ atmosphere to form the Cu_2_Y_2_O_5_ final product.

### 2.4. Synthesis of g-C_3_N_4_ Nanosheets

A thermal oxidative etching approach was used to create the g-C_3_N_4_ samples [27]. Then, the following procedure was employed to synthesize the g-C_3_N_4_ sample: a semi-closed crucible containing 10 g of urea was covered and brought to 550 °C for a timeframe of six hours at a muffle furnace with 10 °C per minute. After that, the resulting, yellow-colored powder was permitted to naturally cool to room temperature before being thoroughly rinsed with ethanol and distilled water. Further, the collected real samples were evaporated to dry in an oven at 60 °C for 12 h and collected.

### 2.5. Synthesis of g-C_3_N_4_/Cu_2_Y_2_O_5_ Composites

The hydrothermal approach was implemented to synthesize the g-C_3_N_4_/Cu_2_Y_2_O_5_ composite. In brief, 260 mg of g-C_3_N_4_ powder was submerged in 60 mL of DI water and ultrasonicated for an hour, and the resulting products were consistently agitated with a magnetic stirrer. The equimolar quantities of prepared Cu_2_Y_2_O_5_ were added to the g-C_3_N_4_ distribution and persistently agitated with a magnetic stirrer for two hours. Afterward, the resulting solution was shifted to a 100 mL Teflon vessel and fixed at 150 °C for 8 h in an autoclave. After that, precipitates were collected in a centrifuge tube and thoroughly rinsed with ethanol and DI water. Finally, the obtained precipitate powder was evaporated to dry for 12 h at 60 °C and used for further application.

### 2.6. Preparation of the GCE/g-C_3_N_4_/Cu_2_Y_2_O_5_ Modified Electrode

**Scheme 1:**
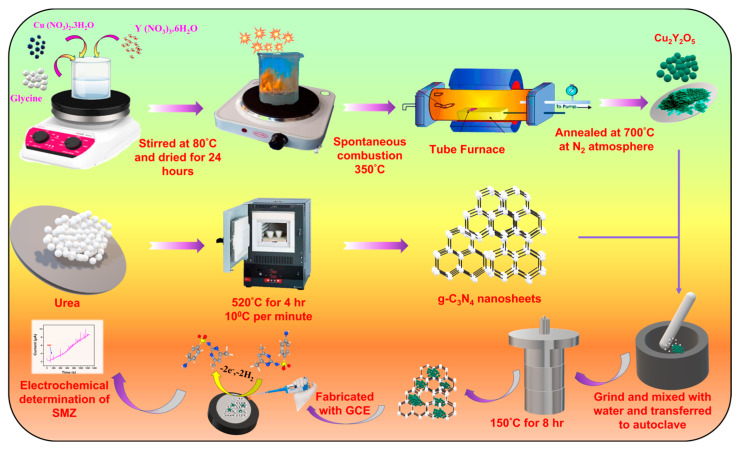
Schematic illustration of GCE/g-C_3_N_4_/Cu_2_Y_2_O_5_ electrode preparation and application to SMZ detection.

To make a glassy carbon electrode (GCE) (surface area 0.07 cm^2^) appear like a mirror, alumina slurry powder was employed to polish it on a micro fabric pad. To get rid of any surface residue, GCE was ultrasonically cleaned multiple times in DI water and ethanol for roughly five minutes after cleaning. After that, the GCE was dried in a hot air oven at 60 °C and rinsed with DI water. The prepared composite was sonicated for 15 min to form a homogenous suspension. Then, the polished, well-cleaned electrode was utilized for the modification of the electrode, and 8 µL of the prepared composite was coated on the GCE electrode by drop casting. After successful coating with the composite, it was dried in the oven at 60 °C to obtain a GCE/g-C_3_N_4_/Cu_2_Y_2_O_5_-modified electrode and used for further electrochemical studies. The detailed GCE/g-C_3_N_4_/Cu_2_Y_2_O_5_-modified electrode preparation procedure is mentioned in Scheme 1.

### 2.7. Preparation of Real Samples

Viability for the GCE/g-C_3_N_4_/Cu_2_Y_2_O_5_-modified electrode was under consideration using honey, milk, and river water, which were further centrifuged at 5000 rpm for about 10 min before the electrochemical analyses. The reaction solution was passed through a Whatman filter paper to remove unfavorable pollutants. The samples of milk, honey, and water were then diluted 50 times with a phosphate-buffered solution for analysis. The very finely commercially ground SMZ powder was weighed and then dissolved in DMF to prepare a 10 mL (0.1 M) solution, which was used for further investigation. To verify real samples, the synthesized g-C_3_N_4_/Cu_2_Y_2_O_5_ composite modified with GCE was used for the electrochemical detection of SMZ.

## 3. Results and Discussion

### 3.1. XRD, Raman, FT-IR, and FESEM Morphology Studies

**Figure 1:**
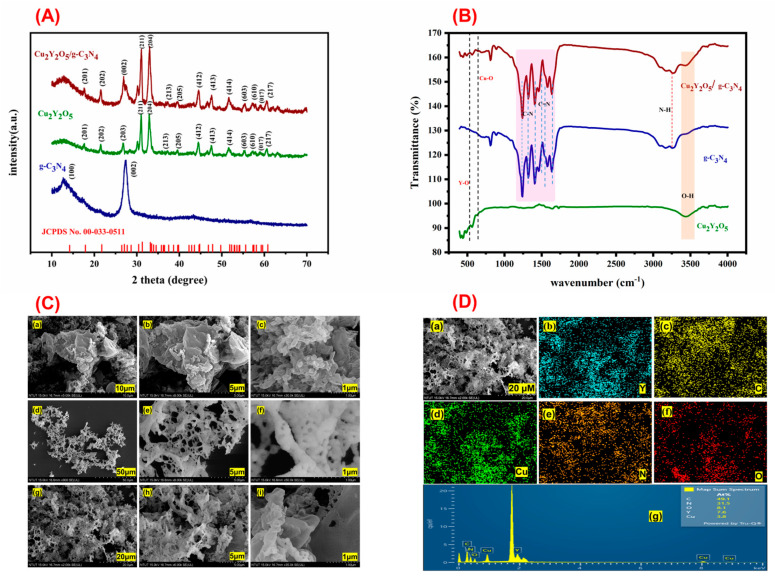
(**A**) XRD patterns of g-C_3_N_4_, Cu_2_Y_2_O_5_, and g-C_3_N_4_/Cu_2_Y_2_O_5_. (**B**) FT-IR spectra of g-C_3_N_4_, Cu_2_Y_2_O_5_, and g-C_3_N_4_/Cu_2_Y_2_O_5_ composite. (**C**) FESEM images (**a**–**c**) g-C_3_N_4_. (**d**–**f**) Cu_2_Y_2_O_5_. (**g**–**i**) g-C_3_N_4_/Cu_2_Y_2_O_5_ composite. (**D**) Elemental mapping region of (**a**) g-C_3_N_4_/Cu_2_Y_2_O_5_ composite. Elemental mapping of (**b**) Y, (**c**) C, (**d**) Cu, (**e**) N, (**f**) O, and (**g**) EDX spectra of g-C_3_N_4_/Cu_2_Y_2_O_5_ composite.

The crystallinity and structure of the synthesized products were analyzed by XRD patterns. Figure 1A explains the XRD patterns of Cu_2_Y_2_O_5_, g-C_3_N_4_ and g-C_3_N_4_/Cu_2_Y_2_O_5_. The XRD pattern of Cu_2_Y_2_O_5_ indicates the crystalline nature with super-accurate distinct peak intensity; likewise, there were no extra diffraction peaks noted. It was matched with a JCPDS card number 00-33-0511. The XRD pattern of g-C_3_N_4_ displays two unique diffraction peaks, at 13.18° and 26.68°. These peaks are related to the crystal lattices (100) and (002); these are linked with the interplanar aromatic stacking (100) planes (JCPDS card number 01-087-1526) [28,29]. In contrast to the bulk g-C_3_N_4_, the power of the (002) peak in g-C_3_N_4_ nanosheets dropped significantly. They show the successful exfoliation of bulk g-C_3_N_4_, as illustrated in Figure 1A. Further, the XRD nature of the g-C_3_N_4_/Cu_2_Y_2_O_5_ composite is displayed in Figure 1A. The noticeable peaks of Cu_2_Y_2_O_5_ were formed by the crystalline plane. The sharp peak of g-C_3_N_4_ was caused by the (002) plane. The crystalline plane overlapped to produce a more dramatic peak than pure g-C_3_N_4_ [28]. However, each single distinct plane was seen in the Cu_2_Y_2_O_5_/g-C_3_N_4_ composite; this demonstrated the successful production of the Cu_2_Y_2_O_5_/g-C_3_N_4_ composite.

Figure 1B displays the FTIR spectrum of the material 1224 cm^−1^, 1316 cm^−1^, and 1399 cm^−1^ showing the C-N bond and the peaks in 1540 cm^−1^, and 1631 cm^−1^. Additionally, the C=N bond of the synthesis material had retained all of the peaks with the inclusion of g-C_3_N_4_. Peaks appeared at 1410 cm^−1^ and 1564 cm^−1^ corresponding to C-O stretching of the carboxylate ion bond. The stretching vibration at 3432 cm^−1^ shows a N-H bond indicating primary amine. The absorption peak at 3725 cm^−1^ shows O-H stretching bonds, which is the alcohol functional group. The absorption peak at 563 cm^−1^ and 531 cm^−1^ shows a Cu-O, Y-O stretching bond.

FE-SEM assessed the surface morphology of the GCE/Cu_2_Y_2_O_5_/g-C_3_N_4_ composite. The link between the layered surfaces of the Cu_2_Y_2_O_5_ and g-C_3_N_4_ composite was observed in the morphological structures of produced materials displayed in Figure 1C. EDX mapping of Cu_2_Y_2_O_5_ and g-C_3_N_4_ was also used to assess the different compositions of the manufactured materials shown in Figure 1D.

### 3.2. Electrochemical Properties

**Figure 2:**
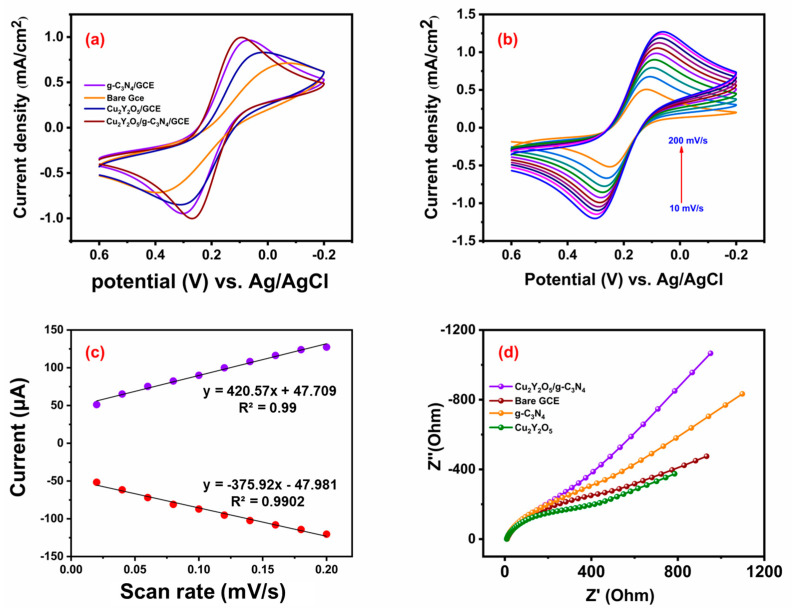
(**a**) CV for bare GCE, GCE/Cu_2_Y_2_O_5_, GCE/g-C_3_N_4_, GCE/Cu_2_Y_2_O_5_/g-C_3_N_4_ in 0.1 M KCl contain 5 mM [Fe(CN)_6_]^3−/4−^ and (**b**) CV studies of Cu_2_Y_2_O_5_/g-C_3_N_4_ at various scan rates (different color indicate for different scan rate). (**c**) calibration plot for potential vs. peak current. (**d**) EIS spectra of bare GCE, Cu_2_Y_2_O_5_/GCE, g-C_3_N_4_/GCE, Cu_2_Y_2_O_5_/g-C_3_N_4_/GCE.

Figure 2 demonstrates an examination of the electrochemical behavior of the as-prepared GCE/g-C_3_N_4_/Cu_2_Y_2_O_5_ electrode. First, the EIS technique was employed to find modifications in the electrochemical properties of the supporting electrolyte and electrode area. A non-destructive electrochemical technique named EIS can quantify variations in the surface attributes of modified electrodes [30]. Higher frequency sections of the EIS spectra reveal a semicircle representing electron-transfer resistance (R_ct_), while low-frequency linearity correlates to electron diffusion. The Nyquist curves of bare GCE, GCE/Cu_2_Y_2_O_5_, GCE/g-C_3_N_4_, and GCE/g-C_3_N_4_/Cu_2_Y_2_O_5_ electrodes in 0.1 M KCl contain 5 mM [Fe(CN)_6_]^3−/4−^ with a stable amplitude of 50 mVs^−1^. The bare GCE electrodes have greater resistance, so it makes the large semicircle diameter, and the R_ct_ values of bare GCE were found to be 446 Ω. Further, the R_ct_ value of Cu_2_Y_2_O_5_-modified GCE shows the R_CT_ values of 383 Ω, which was caused by Cu_2_Y_2_O_5_ increased electrical conductivity. Additionally, the g-C_3_N_4_/GCE electrodes have high conductivity, which explains the existence of g-C_3_N_4_-enhanced electron movement and leads to a high surface area. Remarkably, after the inclusion of g-C_3_N_4_ with Cu_2_Y_2_O_5_, the R_ct_ value decreased to a low 232 Ω; this is probably caused by the increased electron mobility, surface area, and synergistic effect of the Cu_2_Y_2_O_5_ and g-C_3_N_4_ incorporation. Therefore, we have used the GCE/Cu_2_Y_2_O_5_/g-C_3_N_4_ electrode for the SMZ determination due to the high surface area of g-C_3_N_4_ and high electrocatalytic properties of Cu_2_Y_2_O_5_, thus allowing the efficient electrochemical sensing of SMZ.

The electron movement in the bare GCE, GCE/Cu_2_Y_2_O_5_, GCE/g-C_3_N_4_, and GCE/g- C_3_N_4_/Cu_2_Y_2_O_5_ was studied in 0.1 M KCl containing 5 mM [Fe(CN)_6_]^4−/3−^ in the potential between −0.2 and 0.6 vs. Ag/AgCl at a scan rate of 50 mV/s using CV (Figure 2a). CV data discussed the potential difference (E_p_), observed anodic and cathodic peak potentials (E_pa_ and E_pc_), and oxidation and reduction peak current densities (I_pa_ and I_pc_). Consequently, in contrast to other altered and unaltered electrodes, the GCE/g-C_3_N_4_/Cu_2_Y_2_O_5_ had a lower E_p_ and more intriguing redox peak currents caused by a specific electrostatic interaction between the GCE/g-C_3_N_4_/Cu_2_Y_2_O_5_ surface and [Fe(CN)_6_]^4−/3−^ ions. The modified electrodes, like the bare/GCE electrodes, and their electrochemical active surface area (A) for GCE/Cu_2_Y_2_O_5_, GCE/g-C_3_N_4_, and GCE/g-C_3_N_4_/Cu_2_Y_2_O_5_ were calculated using CV in [Fe(CN)_6_]^4−/3−^ solution containing 0.1 M KCl at various sweep scan rate speeds (v) using the Randles–Sevcik equation [31]. In contrast, the scan rates increased, and the current of Cu_2_Y_2_O_5_/g-C_3_N_4_/GCE increased, as seen in Figure 2b. The square root of the graph of the scan rate vs. redox current is shown in Figure 2c. The conductive surface area was to be calculated by Randles–-Sevcik (Equation (1)) for bare GCE, GCE/g-C_3_N_4_, GCE/g-C_3_N_4_/Cu_2_Y_2_O_5_, and GCE/Cu_2_Y_2_O_5_ [32] as follows:

I_p_ = 2.69 × 10^5^ An^3/2^ C D^½^ ν^½^ (1)

where “A” denotes the modified electrode’s surface area, “n” denotes the count of electrons in a reaction, “C” denotes the [Fe(CN)_6_]^4−/3−^ electrolyte concentration in bulk solution (5 mM), “D^1/2^” denotes the square root of the diffusion coefficient, and “v^1/2^” denotes the square root of the scan rate. The active surface area was calculated for bare GCE (0.0273 cm^2^), GCE/Cu_2_Y_2_O_5_ (0.0320 cm^2^), GCE/g-C_3_N_4_ (0.0367 cm^2^), and GCE/g-C_3_N_4_/Cu_2_Y_2_O_5_ (0.0369 cm^2^). From low to high, the peak current intensity is arranged as follows: bare GCE < GCE/Cu_2_Y_2_O_5_ < GCE/g-C_3_N_4_ < GCE/g-C_3_N_4_/Cu_2_Y_2_O_5_. The following Equation (2) was applied to calculate the rates of charge transfer coefficient (Ks) [33].

R_ct_ = RT/(n^2^ F^2^ K_(s)_ C) (2)

In this case, “K_(_s_)_” denotes the transfer of charges rate, “n” denotes the count of electrons, “C” denotes the [Fe(CN)_6_]^−4/−3^ concentration, and “R, T, and F” maintain its unique significance [28,34]. The electrical characteristic results confirm that the GCE/g-C_3_N_4_/Cu_2_Y_2_O_5_ composite has a greater charge transfer rate, a lower ΔE_p_, a faster electron transfer rate, a higher active surface, and a higher current density than GCE/Cu_2_Y_2_O_5_ and GCE/g-C_3_N_4_ electrodes.

### 3.3. Electrochemical Performance Using SMZ Using Cu_2_Y_2_O_5_/g-C_3_N_4_

**Scheme 2:**
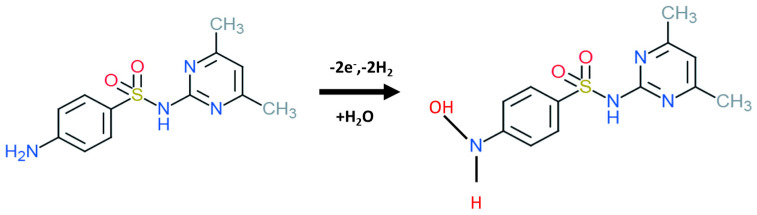
Irreversible electro-oxidation reaction mechanism of SMZ over the GCE/g-C_3_N_4_/Cu_2_Y_2_O_5_ electrode.

**Figure 3:**
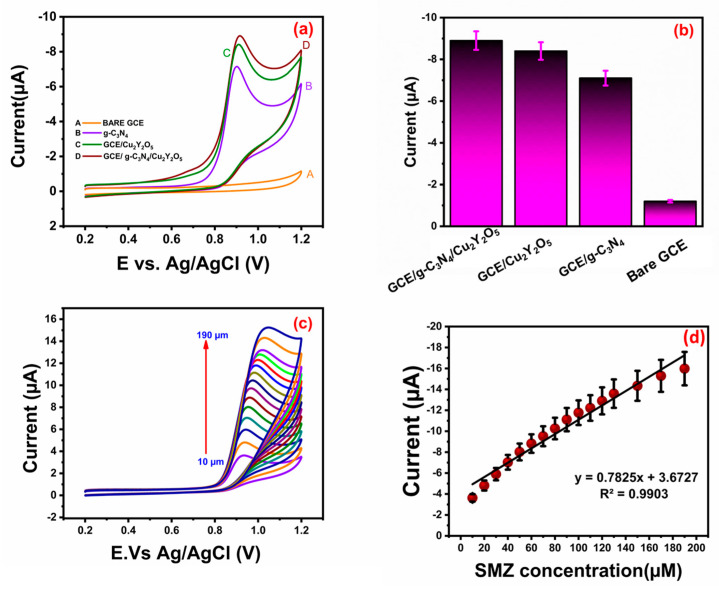
(**a**) Cyclic voltammogram of bare GCE, GCE/g-C_3_N_4_, GCE/Cu_2_Y_2_O_5_, and GCE/g-C_3_N_4_/Cu_2_Y_2_O_5_ in 0.05 M phosphate-buffered solution (pH 7) with 50 μM of SMZ. (**b**) Bar diagram of different electrode responses. (**c**) CV curves of GCE/g-C_3_N_4_/Cu_2_Y_2_O_5_ electrode at different concentrations of SMZ (10–200 μM) (different color indicate for different concentration). (**d**) Correspondence to the calibration plot vs. SMZ concentrations.

The performance of GCE/Cu_2_Y_2_O_5_-, GCE/g-C_3_N_4_-, and GCE/g-C_3_N_4_/Cu_2_Y_2_O_5_-modified electrodes was investigated utilizing SMZ bare electrodes with the help of CV and 0.1 M phosphate-buffered solution (pH 7.0) at 100 mVs^−1^. In addition to 50 μM SMZ, Figure 3a explains the CV profiles of electrodes that have been altered and unaltered. In the beginning, the bare GCE surface did not exhibit any oxidation peak. However, upon the addition of 50 μM SMZ, this observation was confirmed. After the alteration of the bare GCE, GCE/Cu_2_Y_2_O_5_, g-C_3_N_4_, GCE/g-C_3_N_4_/Cu_2_Y_2_O_5_ for the sensing of SMZ with the addition of 50 μM, the spot of the oxidation potential was noted at +0.90 V with this current density (I_pa_) of −7.1 μA/cm^2^, +0.91 V with current density of −8.3 μA/cm^2^, and +0.91 with current density of −8.9 μA/cm^2^, respectively. It was demonstrated that the electrodes’ kinetics of charge transfer to SMZ oxidation were low. The electrodes, SMZ oxidation potential, and oxidation current density were plotted on a graph. Interestingly, the GCE/g-C_3_N_4_/Cu_2_Y_2_O_5_ for SMZ sensing had a considerable oxidation potential of +0.91 V and a greater oxidation current density of around −8.9 μA/cm^2^. This was in comparison to the other electrodes due to the synergistic effect of π-π stacking interplay between SMZ and GCE/g-C_3_N_4_/Cu_2_Y_2_O_5_ electrode, which may enhance the effectiveness of electrocatalytic oxidation for SMZ detection [35]. Additionally, the g-C_3_N_4_/Cu_2_Y_2_O_5_ composite features a layered structure that speeds up the oxidation of SMZ on the GCE/g-C_3_N_4_/Cu_2_Y_2_O_5_ surface, a wide surface area, additional diffusion pathways, high capacitance, good capacitance, and good conductivity. The electrochemical oxidation process of SMZ in the GCE/g-C_3_N_4_/Cu_2_Y_2_O_5_ electrode is displayed in Scheme 2. The irreversible oxidation in the amine group (-NH_2_) in SMZ by 2e^−^ and 2H^+^ movement results in hydroxylamine (-NHOH), as indicated by the oxidation peak [36]. These findings show that the SMZ oxidation reaction is a slow, irreversible process of electron transfer since the reverse scan displays an anodic peak in place of a cathodic peak due to the amine groups oxidation to the -NHOH group and subsequent loss of two electrons. Therefore, we believe that the GCE/g-C_3_N_4_/Cu_2_Y_2_O_5_ composite serves as a workable mediator of electron transport for SMZ detection [37].

When 0.1 M (PH 07) phosphate-buffered solution was used to test the concentration analysis of SMZ, the cathodic peaks increased gradually with the insertion of 10–190 μM. Figure 3. displays the current density vs. SMZ concentration linear plot. Taking this into consideration, fast electron transport and electro-oxidation of SMZ oxidation were demonstrated over the modified GCE/g-C_3_N_4_/Cu_2_Y_2_O_5_ electrode.

### 3.4. Effect of Scan Rate

**Figure 4:**
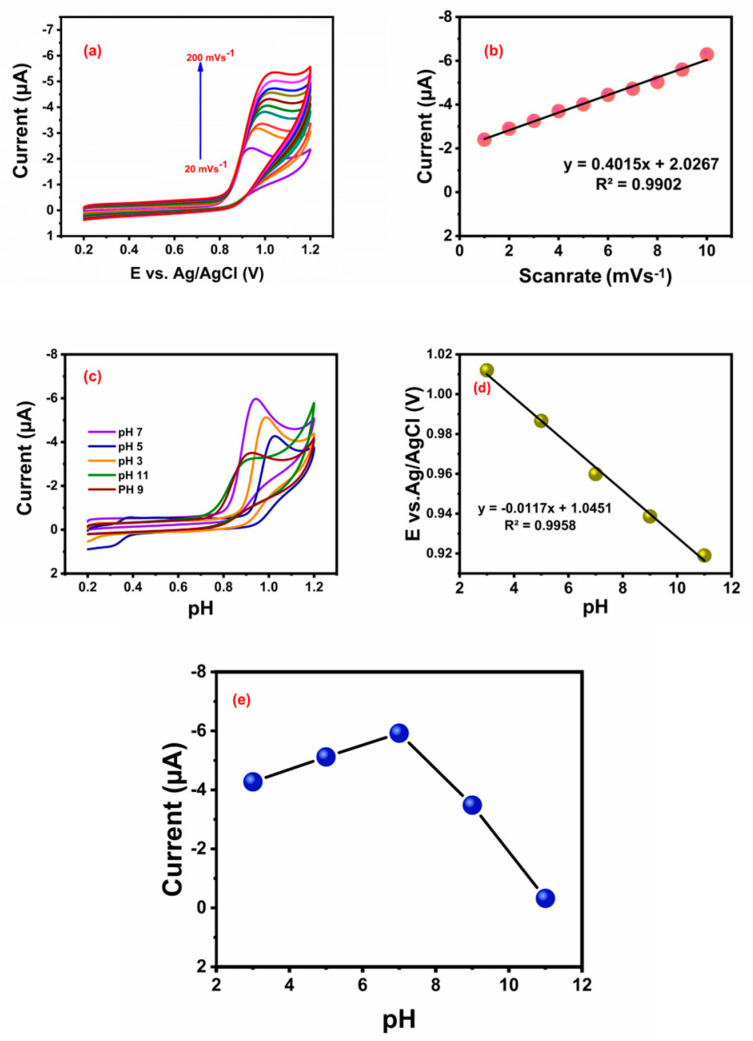
(**a**) CV responses of the electrode GCE/g-C_3_N_4_/Cu_2_Y_2_O_5_ in phosphate-buffered solution with 50 μM of SMZ at various scan rates (different color indicate for different scan rates), (**b**) calibration plot of the scan rate vs. SMZ determination peak current, (**c**) cyclic voltammogram of electrode Cu_2_Y_2_O_5_/g-C_3_N_4_ in phosphate-buffered solution containing 50 μM SMZ at various pH levels (3–11), (**d**) pH vs. E_p_, (**e**) pH vs. I_p_.

In any electrochemical sensor, the scan rate greatly influences the modified electrodes to behave during electrocatalysis. As shown in Figure 4, CVs were tested using g-C_3_N_4_/Cu_2_Y_2_O_5_-modified GCE, changing the scan rate from 20 to 200 mV/s in detail to know about the electro-oxidation of SMZ [38]. The kinetic restriction and mass transfer may have caused the anodic peak potentials to shift over time to the positive side [34]. The anodic current swings according to the square root of the scan rate, which indicates the peak potential moves to the positive side for SMZ oxidation at the GCE/g-C_3_N_4_/Cu_2_Y_2_O_5_ electrode. An electrode diffusion process powers an irreversible reaction with a changed electrode surface. This figure illustrates the linearity range among the square root of the scan rate (mV/s) and the oxidation peak current (I_pa_). The linear equation was found to be I_pa_ (μA) = 0.4015 (mV/s) + 2.0267, with an R^2^ value of 0.9902. Using the E_pa_ and lnν relationship, the Laviron equation (Equation (3)) is used to figure out the number of electrons delivered throughout the irreversible oxidation of SMZ [28,39].

(3) $$E_{pa}=E^{0}+\left(\frac{RT}{\alpha nF}\right)\ln\left(\frac{RTk^{0}}{\alpha nF}\right)+\left(\frac{RT}{\alpha nF}\right)\ln\nu$$

Here “T” is the temperature, “R” is the gas constant, “F” is the Faraday constant, “n” is the number of electrons transported during the chemical process, and “α” is the charge transfer coefficient. The relation led to the calculation of “αn” as 0.8. Because SMZ oxidation is irreversible, it was expected that the coefficient of charge transfer would be 0.5. As a result, “n” was computed as 1.6, meaning that during SMZ oxidation, two electrons took part in the electro-oxidation process on the GCE/g-C_3_N_4_/Cu_2_Y_2_O_5_ electrode.

### 3.5. Effect of pH

An earlier study found that the solution pH level significantly affects the redox activity of electroactive molecules at the proximity [26]. In the SMZ oxidation process, the operating electrolyte’s pH is also vital. Using the CV approach demonstrated above, the electrochemical activity of 50 μM SMZ in 0.1 M phosphate-buffered solution at various pH levels starting from 3 to 11 was examined and is shown in Figure 4. The pH of the electrolytes had an impact on the GCE/g-C_3_N_4_/Cu_2_Y_2_O_5_ electrode determination ability for SMZ sensing. The oxidation peak current intensity rose from 3 to 7, then progressively fell at high pH values. The voltammogram’s sensitivity increased at pH 7. The protonation/deprotonation mechanism of SMZ caused fluctuations in its oxidation response. At pH 7.0, a high anodic current (I_pa_) was achieved. Taking these results into account the complete voltammetric experiment’s initial point was found to be 0.1 M phosphate-buffered solution at pH 7.0 to facilitate future research. The peak potential negative shift showed that pH fluctuations, as well as H^+^ and charge transfer when pH increased, had an impact on it [39]. The scientific relationship between pH and E_pa_ can be understood using the Nernst equation (Equation (4)) that follows.

(4) $$E_{pa}=E^{0}+\frac{0.0591}{n}log\left(\frac{\left({\left(OX\right)}^{a}\right)}{\left({\left(R\right)}^{b}\right)}\right)-\left(\frac{0.0591m}{n}\right)pH$$

Here “m” denotes the number of protons, “n” the number of electrons engaged in the electro-chemical process, “a” and “b” the oxidant and reductant coefficients, and so on [40]. The oxidation peak potential (E_pa_) vs. pH is represented in the calibration plots shown in Figure 4c,d.

### 3.6. Analytical Performance of the Proposed Sensor

**Figure 5:**
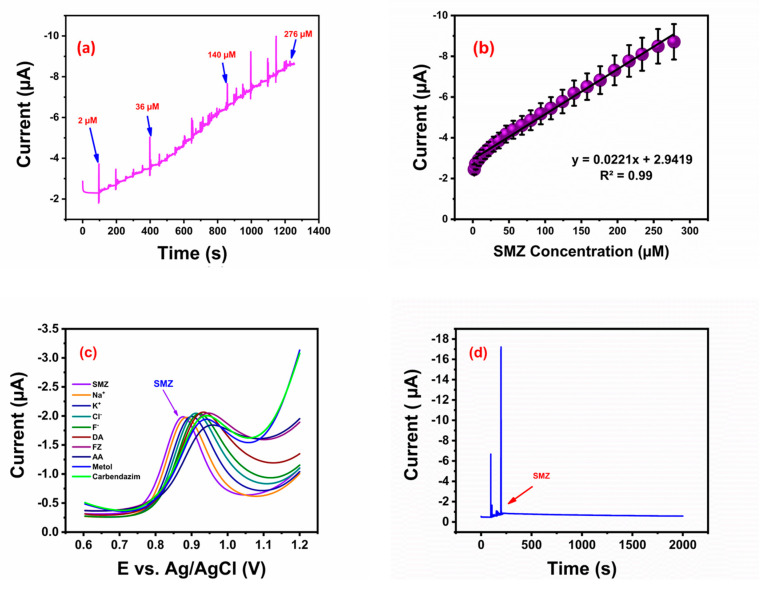
(**a**) Amperometry response for oxidation of SMZ with various concentrations in phosphate-buffered solution (pH 7.0) at RRDE/g-C_3_N_4_/Cu_2_Y_2_O_5_-altered electrode. (**b**) Calibration plot for the peak current vs. concentration. (**c**) Selectivity of the GCE/g-C_3_N_4_/Cu_2_Y_2_O_5_ in phosphate-buffered solution with the addition of a 10-fold concentration of interference molecules by DPV. (**d**) Operational stability studies of the RRD/g-C_3_N_4_/Cu_2_Y_2_O_5_ electrodes.

In this work, the amperometry technique was employed to see if the RRDE/g-C_3_N_4_/Cu_2_Y_2_O_5_ was able to detect SMZ just as well as comparable sensors. Compared to other voltammetric methods, the amperometric approach has higher precision, is more sensitive, and requires less background current [25]. A calibration curve was used to determine the enhanced electrode sensitivity, linear range, and limit of detection (LOD). The g-C_3_N_4_/Cu_2_Y_2_O_5_ composite for modified rotating ring disc electrode (RRDE) amperometric response is depicted in Figure 5a. Following each addition of a different concentration of SMZ into nitrogen-saturated phosphate-buffered solution that is constantly agitated at a rotational speed of 780 rpm with the applied potential held at +0.92 V. Every time SMZ was added, the sensor displayed a crisp amperometric response over a consistent period (50 s). The constant current response of SMZ was obtained at the electrode modified with an RRDE/g-C_3_N_4_/Cu_2_Y_2_O_5_ composite in less than 3 s and demonstrated a quick response to SMZ oxidation. Figure 5b displays the calibration plot of peak current response against the SMZ concentration. Figure 5b shows the equation for the relevant linear regression as follows: I_p(A)_ = 0.0221 × [SMZ (μM)] + 2.9419. It was predicted that the sensing capacity of the sensor was 8.86 μA μM^−1^ cm^−2^ from the slope.

Additionally, the LOD was determined to be 0.23 µm (LOD = 3 Sb/S, where Sb is the standard deviation of the signal and S is the slope value). The constructed sensor, notably, had a linear response from 2 μM to 276 μM. This determined the excellent catalytic oxidation of SMZ at the g-C_3_N_4_/Cu_2_Y_2_O_5_ composite-altered RRDE. Furthermore, because of its excellent electron transport qualities and uniformly electron-rich environment, RRDE/g-C_3_N_4_/Cu_2_Y_2_O_5_ was determined to be a key factor in the oxidation of SMZ based on earlier research. However, Cu_2_Y_2_O_5_ dramatically reduced the SMZ oxidation overpotential, aiding in minimizing power consumption and supporting the prevention of interferences. Furthermore, C_3_N_4_’s large surface area offers more reaction area for its synergy with Cu_2_Y_2_O_5_, making the composite an excellent choice for electrocatalytic reactions. Additionally, improving the electrocatalysis of SMZ was greatly aided by the positive synergistic interaction between g-C_3_N_4_ and Cu_2_Y_2_O_5_ [28,35].

Nanomaterials have been used in previous works in electroanalytical methods to modify the electrodes to determine the SMZ samples (Table 1). In comparison, a two-electrode sensor system for rapid detection of sulfonamides by applying the Nafion-carboxyl multiwalled carbon nanotube powder microelectrode by B. He et al. found a limit of detection (LOD) of 8.94 µM [41]. A novel electroanalytical assay for sulfamethazine determination in food samples based on conducting polymer composite-modified electrodes by Y. L. Su et al. found a limit of detection of 0.16 µM [41]. Moreover, magnetic hyper-crosslinked polystyrene used for sulfonamides from water and milk samples before their HPLC determination by V.V. Tolmacheva et al. found a LOD of 0.25 µM [42]. T.N. Rao et al.’s diamond electrodes used for the detection of sulfa drug determination by amperometric and HPLC detection found a LOD of 0.05 µM [43]. The occurrence and removal of antibiotics, hormones, and several other pharmaceuticals in wastewater treatment plants of the largest industrial city of Korea by Behera et al. found a LOD of 0.055 µM [44]. Development of an electrochemical sensor for the determination of antibiotic sulfamethazine in cow milk using graphene oxide decorated with Cu-Ag core–shell nanoparticles by A. Feizollahi et al. found a LOD of 0.46 [42].

### 3.7. Selectivity, Repeatability, Reproducibility, and Storage Stability Studies

One of the most crucial features of the proposed sensor is selectivity. Multiple biological species coexist for real-time utilization purposes to determine whether SMZ sensing was hampered by the sensing platform. The effects of the relationship induced by the electrochemical behavior of the proposed sensor signal were examined. The GCE/g-C_3_N_4_/Cu_2_Y_2_O_5_ electrode selectivity was measured, in this case, using the DPV technique when there is a 10-fold concentration of frequently hindering biomolecules like metal, ascorbic acid, dopamine, and carbendazim, as well as common cations like sodium and ammonium, as well as anions like chloride and fluoride. This outcome shows the GCE/g-C_3_N_4_/Cu_2_Y_2_O_5_-modified electrode has great accurate sensitivity towards SMZ. Unexpectedly, the oxidation capability of SMZ remained unaltered after the insertion of the biomolecules claimed earlier. So, the observation suggests that the impact of chemical intervenors was negligible.

**Figure 6:**
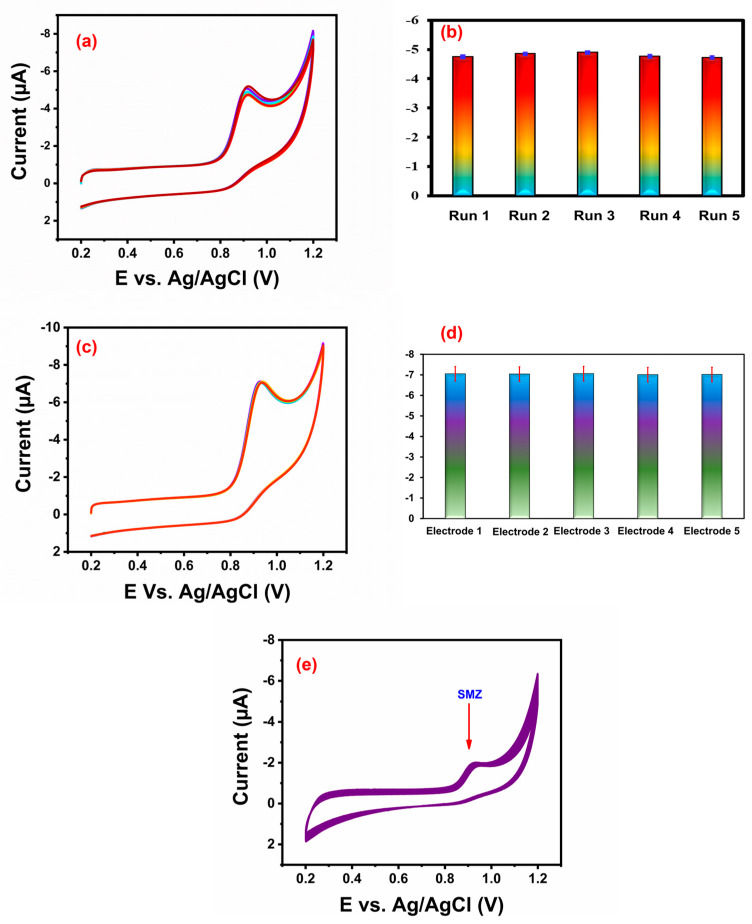
(**a**) Repeatability test of electrode GCE/g-C_3_N_4_/Cu_2_Y_2_O_5_ in the presence of SMZ and (**b**) Bar diagram of GCE/g-C_3_N_4_/Cu_2_Y_2_O_5_ electrode with different segments. (**c**) The reproducibility test of GCE/g-C_3_N_4_/Cu_2_Y_2_O_5_ electrode in the presence of SMZ and (**d**) Bar diagram of different modified GCE/g-C_3_N_4_/Cu_2_Y_2_O_5_ electrodes. (**e**) Cyclic stability of GCE/g-C_3_N_4_/Cu_2_Y_2_O_5_ electrode.

The selective oxidation towards SMZ might be quickened by a favorable π-π stacking contact that occurs among the SMZ molecule and the Cu_2_Y_2_O_5_/g-C_3_N_4_ composite. Furthermore, the hindering molecules’ oxidation potential was in contrast to the range of oxidation of the SMZ. Therefore, it does not interrupt the SMZ oxidation current density [35]. Additionally, the sensor’s stability, repeatability, and reproducibility were examined for SMZ oxidation and are displayed in Figure 6a–d. This is why the GCE/g-C_3_N_4_/Cu_2_Y_2_O_5_ electrodes were prepared five times independently to be able to assess the reliability of the sensors. The experiment of testing 50 μM SMZ at the GCE/g-C_3_N_4_/Cu_2_Y_2_O_5_ electrode multiple times was employed to examine the sensor’s repeatability and stability. Long-term stability was assessed after storing it for approximately 1, 7, 14, 21, and 30 days. The sensors kept 93.8% of the responses. The sensors showed excellent reproducibility in the same procedure. Additionally, Figure 6e shows the long-usage stability of the GCE/g-C_3_N_4_/Cu_2_Y_2_O_5_ electrode utilizing continuous CV monitoring for 100 segments in 0.1 M phosphate-buffered solution. During this period, the electrode current responsiveness only dropped by about 12%. The outcomes verified that, following analyses and extended periods of storage, the GCE/g-C_3_N_4_/Cu_2_Y_2_O_5_ had demonstrated outstanding stability. Additionally, 50 s intervals were used to spike 10 μM and 20 μM of SMZ in the amperometry stability investigation, determined up to 2000 s. After stability testing, the prepared sensor maintained 98.79% of its initial efficiency.

### 3.8. Real Sample Analysis

**Scheme 3:**
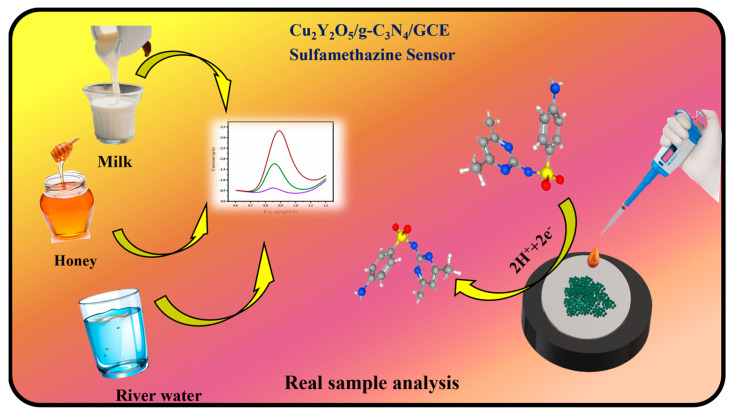
Real sample analysis and reaction mechanism of the proposed GCE/g-C_3_N_4_/Cu_2_Y_2_O_5_ electrode sensor.

Assessing the potential threats that pharmaceutical toxicity may pose to human health and the environment requires real sample monitoring. It has contributed to the practical application of the g-C_3_N_4_/Cu_2_Y_2_O_5_ composite-modified GCE in SMZ determination in real sample analysis. As previously noted, real samples are typically free of SMZ. Examples of such samples are milk, blood serum, poultry, fish, meat, honey, water, etc. [3,28]. Hence, real samples were added with a predetermined amount of SMZ utilizing standard addition methods (Scheme 3).

**Figure 7:**
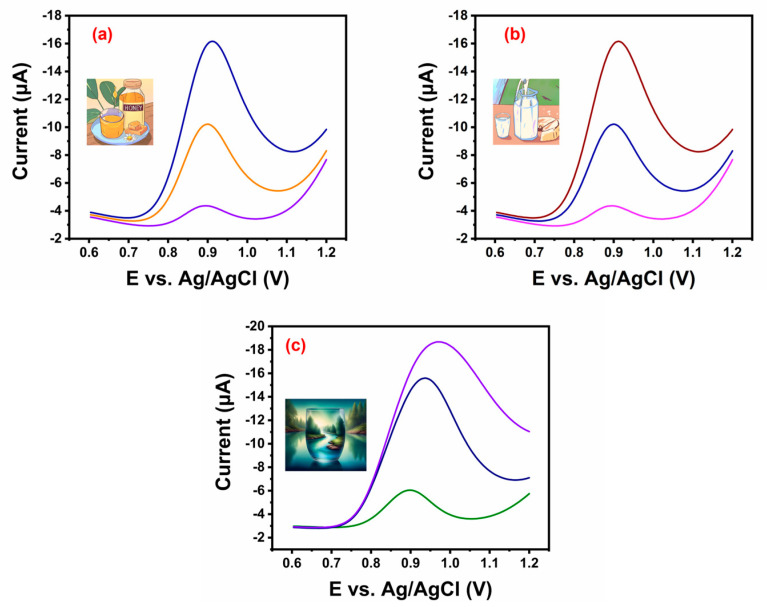
The determination of SMZ in (**a**) milk, (**b**) honey, and (**c**) river water samples (different color indicate for different concentration).

The GCE/g-C_3_N_4_/Cu_2_Y_2_O_5_ electrode was evaluated for its practicality in identifying SMZ in real samples of cow milk, honey, and Tamsui river water using the DPV approach (Figure 7a–c). The range of SMZ recovery rates was 92–98%. These results demonstrated that the GCE/g-C_3_N_4_/Cu_2_Y_2_O_5_ electrode was used with satisfactory results to analyze SMZ in real samples. The recovery percentage findings are displayed in Table 2. Even when milk, honey, or Tamsui river water contains other interfering molecules, the DPV approach uniquely finds SMZ. Because the SMZ detection peak potential and peak current were not affected by other interfering molecules, such as anions, cations, metal ions, and other biomolecules, the developed GCE/g-C_3_N_4_/Cu_2_Y_2_O_5_ electrode is the ideal match for the accurate and successful sensing of SMZ from the real sample in the presence of other interfering molecules. According to the real sample analysis, the GCE/g-C_3_N_4_/Cu_2_Y_2_O_5_ electrode shows good sensing feasibility for SMZ detection.

## 4. Conclusions

It was discovered that the electrode fabricated with GCE/g-C_3_N_4_/Cu_2_Y_2_O_5_ was effective in the electrocatalytic oxidation of SMZ. About the electro-oxidation of SMZ, it was noted that the GCE/g-C_3_N_4_/Cu_2_Y_2_O_5_ electrode is a cutting-edge electrocatalyst. The Cu_2_Y_2_O_5_, g-C_3_N_4_, and g-C_3_N_4_/Cu_2_Y_2_O_5_ composites were examined by XRD, Raman, FT-IR, FE-SEM, and EDX studies. Cyclic voltammetry and amperometry methods were employed for the selective use of a GCE/g-C_3_N_4_/Cu_2_Y_2_O_5_ electrode for the electro-oxidation of SMZ. Additionally, important kinetic characteristics of GCE/g-C_3_N_4_/Cu_2_Y_2_O_5_ electrode rate constant (K_(s)_) and effective surface area were calculated. The GCE/g-C_3_N_4_/Cu_2_Y_2_O_5_ can be performed towards the detection of SMZ from a line range up to 276 μM, with a LOD of 0.23 μM and linear range 2 to 276 µM by the amperometry method. It was further confirmed that the GCE/g-C_3_N_4_/Cu_2_Y_2_O_5_ electrode is repeatable. Over 30 days, the GCE/g-C_3_N_4_/Cu_2_Y_2_O_5_ electrode showed good stability, repeatability, and reproducibility. Lastly, the increased precision of the g-C_3_N_4_/Cu_2_Y_2_O_5_ fabricated electrode effectively made use of SMZ detection in food and water samples. Another benefit is that the proposed sensor exhibits a low detection limit with the highest sensitivity and will be used to build commercial electrodes in the future.

## Figures and Tables

**Table 1:** The proposed GCE/g-C_3_N_4_/Cu_2_Y_2_O_5_ sensor was compared with previously reported determination techniques.

Technique	Electrodes	Linear Range (µM)	LOD (μM)	References
CV	GCE/rGO/AuNPs	-	-	[43]
i-t	PME/N-MWCNT	10–100	8.94	[44]
SWV	SPCE/PEDOT/MnO_2_	1.0–500	0.16	[40]
MSPE-HPLC-i-t	GCE	2.0–200	0.25	[39]
HPLC-i-t	BDD	0.05–500	0.05	[45]
DPV	AuNSs/LIPG	0.4–100	0.055	[46]
i-t	SN/rGO/GCE	0.009–128	0.006	[47]
SWV	Cu-Ag core-shell/GO/GCE	10–1000	0.46	[42]
CV	Car/MWCNTs/GCE	0.50–110	0.02–0.09	[48]
i-t	Cu_2_Y_2_O_5_/g-C_3_N_4_/GCE	2–276	0.23	**This work**

**Table 2:** Determination of SMZ levels in the milk, honey, and river water samples using GCE/g-C_3_N_4_/Cu_2_Y_2_O_5_ electrode.

Sample	Added (µM)	Found (µM)	Recovery %
Milk	10	9.37 (4.35 µA)	93%
	20	19.5 (10.2 µA)	97%
	30	28.2 (16.1 µA)	96%
Honey	10	9.6 (4.9 µA)	96%
	20	19.77 (10.6 µA)	98%
	30	29.1 (16.7 µA)	97%
River water	10	9.2 (6.6 µA)	92%
	20	18.92 (15.5 µA)	94%
	30	29.03 (18.7 µA)	96%

## Data Availability

Data will be provided by request.
